# First complete mitochondrial genome of *Armillifer moniliformis* (Pentastomida: Porocephalida) isolated from a human case in Northern Thailand: comparative and phylogenetic analyses

**DOI:** 10.1007/s00436-025-08516-x

**Published:** 2025-06-27

**Authors:** Thanapat Pataradool, Padet Siriyasatien, Woraporn Sukhumavasi, Saruda Tiwananthagorn, Chusana Suankratay, Kanok Preativatanyou

**Affiliations:** 1 Medical Sciences Program, Graduate School, Faculty of Medicine, Chulalongkorn University, Bangkok, 10330 Thailand; 2 Department of Parasitology, Faculty of Medicine, Chulalongkorn University, Bangkok, 10330 Thailand; 3 Center of Excellence in Vector Biology and Vector-Borne Disease, Faculty of Medicine, Chulalongkorn University, Bangkok, 10330 Thailand; 4 Parasitology Unit, Department of Pathology, Faculty of Veterinary Science, Chulalongkorn University, Bangkok, 10330 Thailand; 5 Feline Infectious Disease and Health for Excellence Research Unit, Microbial Food Safety and Antimicrobial Resistance Research Unit, Center of Excellence in Animal Vector-Borne Diseases, Chulalongkorn University, Bangkok, 10330 Thailand; 6 Faculty of Veterinary Medicine, Chiang Mai University, Chiang Mai, 50100 Thailand; 7 Research Center of Producing and Development of Products and Innovations for Animal Health and Production, Faculty of Veterinary Medicine, Chiang Mai University, Chiang Mai, 50100 Thailand; 8 Division of Infectious Diseases, Department of Medicine, Faculty of Medicine, Chulalongkorn University, Bangkok, 10330 Thailand

**Keywords:** *Armillifer*, Pentastomids, Zoonosis, Mitogenome, Phylogenetics, Northern Thailand

## Abstract

Pentastomiasis, a zoonotic parasitic disease caused by pentastomids, has been increasingly documented in Thailand. Historically, morphological identification has been the only diagnostic method, but it is inadequate due to morphological similarities between larval stages and limited taxonomic keys. To overcome this, molecular diagnosis is required. However, the DNA sequence information of these neglected parasites remains largely unknown. In this study, pentastomid larvae were collected from the greater omentum of a patient in Northern Thailand. The species of pentastomid larvae was molecularly identified as *Armillifer moniliformis*, using PCRs targeting nuclear and mitochondrial regions, followed by Sanger sequencing. Nanopore sequencing was employed to characterize the complete mitochondrial genome of *A. moniliformis*, revealing a total length of 16,367 bp with 37 mitochondrial genes, including 13 protein-coding genes, 22 tRNA genes, and two rRNA genes, as well as one non-coding region. The entire mitogenome is highly AT-rich (62.1%), with nucleotide skews indicating compositional bias between strands. The non-coding region (2616 bp) lies between the tRNA-Leu^CUN^ and tRNA-Ser^UCN^ genes and comprises three repetitive segments. The third repetitive segment is the longest (1659 bp) and has the highest A + T content of 72.2%, likely serving as a regulatory element related to DNA replication. Comparative mitogenome analysis revealed *cox1* as the most conserved gene, while *atp6*, *atp8*, and *nad6* exhibit high variability among species. Codon usage analysis indicated that two codon families (Leu1 and Ile) are used more frequently than others, suggesting that these branched-chain hydrophobic amino acids are predominant in transmembrane proteins and underscoring the critical role of the mitochondrion in energy production. Based on single gene (*cox1*) and concatenated gene-based phylogenetic approaches, *A. moniliformis* clustered closely with other *Armillifer* species, with all pentastomids placed taxonomically in the subphylum Crustacea. Mitogenomic phylogenetics in this study demonstrates greater topological precision than *cox1* phylogenetics. In conclusion, this study revealed the first complete mitochondrial genome of *A. moniliformis*, providing us with novel molecular markers that could effectively facilitate species diagnosis, treatment monitoring, and epidemiological surveillance of pentastomiasis.

**Supplementary Information:**

The online version contains supplementary material available at 10.1007/s00436-025-08516-x.

## Introduction

Pentastomes, or pentastomids, are arthropod parasites that infect the lungs and respiratory tract of reptiles and other vertebrates (Riley 1986). These parasites are distributed worldwide, particularly in tropical and subtropical countries such as Africa, China, and Southeast Asia. To date, pentastomids have been systematically classified into four orders: Cephalobaenida, Porocephalida, Raillietiellida, and Reighardiida (Christoffersen and De Assis 2013). Among these, two major genera of Porocephalida are *Linguatula* and *Armillifer*. *Linguatula* has carnivores as definitive hosts, while *Armillifer* has snakes as definitive hosts, with both genera using mammals as intermediate hosts. These two genera are also known to be of zoonotic importance, with humans acting as an accidental intermediate host (Tappe and Warrell 2020).

*Linguatula* spp. have been reported in humans and animals with global distribution in Europe, the Middle East, Africa, and the Americas. In contrast, *Armillifer* spp. are distributed in certain regions, including *A. agkistrodontis* in China, *A. armillatus* and *A. grandis* in Africa, and *A. moniliformis* in Southeast Asia (Chen et al. 2010). For *Armillifer* spp., intermediate mammalian hosts can acquire these parasites by ingesting undercooked snake meat contaminated with infective embryonated eggs. The ingested larva of these parasites develops into a nymph, which causes visceral pentastomiasis by encysting in the viscera, such as the omentum, intestinal wall, and liver (Meyers and Neafie 2011). Given the morphological similarity of the larval stage between species, it is difficult to provide an accurate species diagnosis (Tappe and Buttner 2009).

The definitive diagnosis of most parasitic diseases is traditionally based on morphological species identification of the causative parasite. However, this method still has limitations, especially in cases of inadequate or degenerative specimens, morphological similarities of the immature stage among closely related species, and the unavailability of taxonomic keys. To overcome these, molecular techniques have been applied to establish the definitive diagnosis of several infectious diseases that cannot be diagnosed by microscopy (Ndao 2009). Currently, mitochondrial DNA has been widely used as a molecular marker for parasite species identification and evolutionary studies due to its high copy number with interspecific genetic variation (Chan et al. 2021; Galtier et al. 2009). Accordingly, DNA-based identification is likely advantageous as an alternative tool for diagnosing pentastomiasis.

In Thailand, there has been an increasing number of pentastomiasis cases in which immature larvae are intraoperatively found in various human and animal tissues. However, all parasite specimens collected from these cases were solely morphologically identified at the family or genus level as Porocephalidae and *Armillifer*, respectively, without species confirmation (Manunpichu 1981; Sambhavaphol and Orparyoon 1985; Sarasas et al. 1976; Sirisumpan et al. 1968; Tescharoen and Laohapand 1986; Thamprasert 1992; Tiewchaloren et al. 1999). Furthermore, the species diversity of pentastomids in the country remains largely unknown. As such, a molecular diagnosis would enable us to identify the causative parasites, facilitate the investigation of the potential causes and associated risk factors, enhance our understanding of the parasite diversity, and support the development of prevention and control strategies for this neglected zoonotic disease.

In this research, we aim to (i) characterize the complete mitochondrial genome of *A. moniliformis* collected from a Thai patient in Northern Thailand, (ii) analyze its mitogenome comparatively with other pentastomid species, and (iii) investigate the phylogenetic relationships across the phylum Arthropoda. Here, we utilize a long-read Nanopore sequencing technology with an enrichment-free approach to generate high-coverage and high-accuracy sequencing data. Furthermore, comparative and phylogenetic analyses from this study will provide a more complete understanding of the identification, systematics, and evolution of *Armillifer* and other pentastomids.

## Materials and methods

### Sample collection and DNA extraction

An excised omental sample containing several encysted *Armillifer* larvae was obtained from a patient in Phitsanulok Province, Northern Thailand, who was recently diagnosed with pentastomiasis. The fresh sample was transported in a 0.9% sodium chloride solution and dissected in a sterile Petri dish to isolate the parasitic larvae. Ten larval specimens were washed thrice with phosphate-buffered saline and immediately preserved in 70% ethanol. Total genomic DNA (gDNA) was extracted from the whole body of the pentastomid larva using a DNeasy Blood & Tissue Kit (Qiagen, Hilden, Germany) following the manufacturer’s protocol. The concentration and purity of the DNA were measured by a NanoDrop 2000c spectrophotometer (Thermo Fisher Scientific, MA, USA). The extracted DNA from each sample was kept at –20 °C until use.

### PCR amplification and DNA sequencing

To identify the pentastomid species, gDNA samples were employed as a template for conventional PCR amplification targeting several regions, namely cytochrome c oxidase subunit I (*cox1*), NADH dehydrogenase subunit 5 (*nad5*), internal transcribed spacer 2 (ITS2), small subunit ribosomal RNA (18S rRNA), and large subunit ribosomal RNA (28S rRNA). Each PCR reaction was carried out in a total volume of 25 µl containing 12.5 µl of 2× KAPA HiFi HotStart ReadyMix (Roche, Basel, Switzerland), 2 µl of gDNA, 1 µl of each 10 µM primer, and 8.5 µl of nuclease-free water, with thermocycling conditions as previously described (Table S1). The amplification products were verified by 1.5% (*w/v*) agarose gel electrophoresis with ethidium bromide staining. The stained gels were visualized under the GelDoc Go Imaging System (Bio-Rad, CA, USA).

Each positive amplicon was cloned into the pGEM^®^-T Easy Vector (Promega, WI, USA) and chemically transformed into competent *Escherichia coli* DH5α cells. Transformants were cultured at 37 °C for 16–18 h on Luria–Bertani (LB) agar plates containing ampicillin, IPTG, and X-Gal for blue-white colony selection. White colonies on each plate were selected and confirmed for positive inserts by colony PCR. Five positive colonies were inoculated overnight into LB broth supplemented with ampicillin. Recombinant plasmids were extracted using the Invisorb^®^ Spin Plasmid Mini Kit (STRATEC, Birkenfeld, Germany) and subjected to Sanger sequencing using T7 and SP6 promotor primers serviced by Macrogen, Inc. (Seoul, Korea).

### Species identification

The resulting sequence electropherograms were visualized using Unipro UGENE version 51.0 (Okonechnikov et al. 2012) to trim out the flanking plasmid vector sequences and to assemble between forward and reverse sequences. Multiple sequence alignment of five assembled sequences from each target region was performed to generate a consensus sequence. The consensus sequence of each gene region was compared with the GenBank references using the nucleotide BLAST (BLASTn) search tool (https://blast.ncbi.nlm.nih.gov/Blast.cgi) (Johnson et al. 2008) to identify the species of the pentastomid. All consensus sequences obtained in this study were deposited in GenBank.

### Mitochondrial genome sequencing by nanopore MinION^®^ flow cell

Five gDNA samples were pooled and cleaned up using Agencourt AMPure XP beads (Beckman Coulter, CA, USA) and quantified with Qubit™ 4 fluorometer using the Qubit™ dsDNA High Sensitivity Assay Kit (Thermo Fisher Scientific, MA, USA) prior to nanopore sequencing to construct a complete mitogenome of the identified pentastomid. A Rapid Barcoding Kit V14 (SQK-RBK114.24; Oxford Nanopore Technologies (ONT), Oxford, UK) was used for DNA library preparation following the manufacturer’s instructions. The library was subsequently sequenced using MinION^®^ R10.4.1 flow cell for 4 h. Dorado version 0.9.1 was used for super-accuracy base calling with dna_r10.4.1_e8.2_400 bps_sup@v5.0.0 model, demultiplexing, and adaptor-barcode trimming. Low-quality sequences were filtered out using Chopper version 0.9.0 (De Coster and Rademakers 2023), and only high-quality sequences with a quality score of at least 20 were used for mitogenome assembly.

### Mitogenome assembly and annotation

ONT-sequenced reads were assembled using a bioinformatics pipeline for a complete mitochondrial genome. Previously filtered reads were subjected to de novo assembly using Miniasm version 0.3-r179 (Li 2016). Generated contigs with a circular configuration and a size between 16,000 and 17,000 bp were selected to be mapped against reference pentastomid mitochondrial sequences available in GenBank using BLASTn. The contig with the highest percent identity to those of other *Armillifer* species was then selected and error-polished with filtered reads using Medaka version 2.0.1 (https://github.com/nanoporetech/medaka) to generate a consensus mitogenome sequence.

The obtained consensus sequence was subsequently annotated using MitoZ version 3.6 (Meng et al. 2019) based on the mitochondrial genetic code of invertebrates. Pairwise comparisons of annotated protein-coding gene (PCG) sequences and the reference mitochondrial genomes of *A. armillatus* (AY456186), *A. agkistrodontis* (KX686568), and *A. grandis* (KY914472) were conducted by multiple alignment using fast Fourier transform (MAFFT) version 7.526 (Katoh and Standley 2013) with default parameters to confirm the accuracy of the annotation. The missing tRNA genes were localized using MITOS2 version 2.1.9 (Bernt et al. 2013). The boundaries of rRNA genes were determined by flanking tRNA genes and a PCG as previously described in other *Armillifer* spp. (Lavrov et al. 2004; Li et al. 2016). The small subunit (12S) rRNA gene (*rrnS*) is located between tRNA-Leu^CUN^ (*trnL*_*1*_) and tRNA-Val (*trnV*), whereas the large subunit (16S) rRNA gene (*rrnL*) is positioned between *trnV* and *nad1*. The repeat regions in the non-coding region (NCR) were detected by Tandem Repeats Finder version 4.09 (Benson 1999). The annotated mitogenome of *A. moniliformis* was visualized using Proksee (https://proksee.ca) (Grant et al. 2023) and submitted to the GenBank database.

### Validation of mitochondrial genome by Sanger sequencing

To confirm the accuracy of the mitochondrial sequence generated by ONT, six regions, including four PCGs (*cox1*, *cox2*, *cytb*, and *nad5*), *rrnL*, and NCR, were amplified with newly designed primers (Table S2). PCR products were verified by 1.5% (*w/v*) agarose gel electrophoresis with staining and visualization as previously described. Each amplicon was purified using a QIAquick PCR Purification Kit (Qiagen, Hilden, Germany) and subjected to bidirectional DNA sequencing by Macrogen, Inc. (Seoul, Korea). The obtained chromatogram of each amplicon was verified for trimming low-quality signals and then assembled. Pairwise alignment of the resulting Sanger sequences and the previously generated mitochondrial sequence was performed to identify dissimilar nucleotide bases, and the correct nucleotide bases were manually edited based on the Sanger sequencing.

### Comparative mitogenome analysis

The complete mitogenome of *A. moniliformis* obtained from this study was compared with those of *A. armillatus* (AY456186), *A. agkistrodontis* (KX686568), *A. grandis* (KY914472), *Linguatula arctica* (MN792849), and *L. serrata* (MG951756) in terms of gene content, length, and order, as well as nucleotide composition, codon usage, and relative synonymous codon usage (RSCU) using MEGA version 11 (Tamura et al. 2021). The compositional skew was computed using the following formulas: AT skew = (A – T)/(A + T) and GC skew = (G – C)/(G + C) as described elsewhere (Perna and Kocher 1995). The nucleotide diversity of PCGs and rRNAs was also calculated using DnaSP version 6.12.03 (Rozas et al. 2017) through a sliding window with a window size of 250 bp and a step size of 25 bp. The RSCU value of a codon was defined as the observed frequency of that codon divided by its expected frequency, which was under the assumption of equal utilization of synonymous codons for the specific amino acid it encodes (Sharp and Li 1986). The codon with an RSCU value of 1 is considered to have no codon usage bias, while RSCU values greater than 1.6 and less than 0.6 indicate overrepresentation and underrepresentation of codons, respectively (Wong et al. 2010).

### Phylogenetic analysis

The complete mitochondrial genome of *A. moniliformis* retrieved from this study and other 28 representative mitogenome sequences from the phylum Arthropoda with *Hypsibius dujardini* (FR749884) as an outgroup (Table S3) were enrolled for phylogenetic analysis. We performed a comparative phylogenetic study using nucleic acid sequence alignment of 13 PCGs and the *cox1* gene. Sequence alignment for each PCG was performed separately using TranslatorX version 1.1 (Abascal et al. 2010) with the MAFFT algorithm and subsequently concatenated into a single dataset. Phylogenetic trees were constructed using IQ-TREE version 2.3.6 (Minh et al. 2020) with the maximum likelihood (ML) method. Specifically, an optimal partitioning strategy based on 13 partitions (i.e., 13 PCGs) was applied for the concatenated dataset. The selection of the most suitable substitution model was executed automatically by ModelFinder (Kalyaanamoorthy et al. 2017) with 1000 ultrafast bootstraps calculated using UFBoot (Minh et al. 2013). Phylogenetic trees were then rendered using FigTree version 1.4.4 (http://tree.bio.ed.ac.uk/software/figtree/).

## Results

### Molecular identification of *A. moniliformis*

Mitochondrial (*cox1* and *nad5*) and nuclear (ITS2, 18S rRNA, and 28S rRNA) genes were successfully amplified from extracted gDNA samples, resulting in amplicon sizes of 707, 358, 544, 1842, and 1367 bp, respectively (Fig. S1). Plasmid sequencing and BLASTn analysis showed that all amplicons except 28S rRNA were genetically similar to *A. moniliformis* references, with a high percentage of identity ranging from 97.7 to 100% (Table S4). However, our 28S rRNA sequence showed only 86.5% similarity to that of *Porocephalus* sp. (MT387200), possibly due to the unavailability of the *Armillifer* 28S rRNA sequence in the database. Therefore, we identified the parasite samples as *A. moniliformis*. The *cox1*, *nad5*, ITS2, 18S rRNA, and 28S rRNA consensus sequences were deposited in the GenBank database under the accession numbers PP272417, PP273415, PP273236, PV124096, and PV132650, respectively.

### Mitogenome assembly and annotation of *A. moniliformis*

**Fig. 1:**
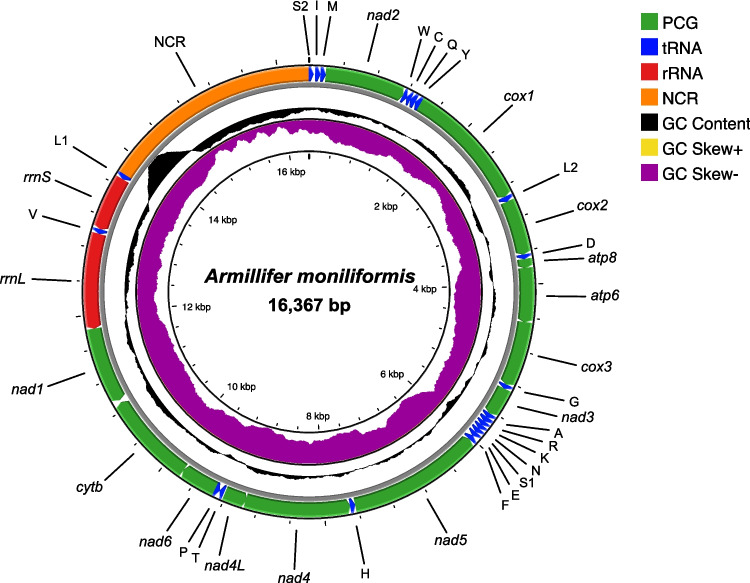
Complete mitochondrial genome of *A. moniliformis*. Different colors represent features of the mitochondrial genome: PCG (green), tRNA (blue), rRNA (red), NCR (orange), GC content (black), GC skew+ (yellow), and GC skew− (purple). Transfer RNA (tRNA) genes are labeled following the IUPAC-IUB single-letter amino acid codes

Nanopore sequencing was employed to generate high-throughput data for the assembly of a complete mitochondrial genome of *A. moniliformis*. After filtering out low-quality reads, 119,518 sequenced reads with a quality score of ≥ 20 were retained, yielding a total base count of 572 Mb for mitogenome assembly. Subsequent bioinformatic analysis revealed that the complete mitochondrial genome of *A. moniliformis* is 16,367 bp in length exhibits a circular configuration and contains a typical set of 37 metazoan mitochondrial genes, including 13 PCGs, 22 tRNA genes, and two rRNA genes. The genes were distributed unequally between the heavy (H) and light (L) strands. Fourteen genes, including four PCGs (*nad5*, *nad4*, *nad4L*, and *nad1*) and eight tRNA genes (tRNA-Cys, tRNA-Gln, tRNA-Tyr, tRNA-Phe, tRNA-His, tRNA-Pro, tRNA-Val, and tRNA-Leu^CUN^), as well as two rRNAs (*rrnL* and *rrnS*), were encoded on the H-strand. The remaining 23 genes were encoded on the L-strand. Additionally, a long NCR spanning 2616 bp was identified between tRNA-Leu^CUN^ and tRNA-Ser^UCN^. Using the Tandem Repeats Finder, the NCR of *A. moniliformis* comprised three repetitive segments, with an average A + T content of 62.3%. The first segment was 692 bp long, featuring 15.7 tandem repeats and an A + T content of 36.6%, while the second had 2.2 tandem repeats, each 13 bp in length. The third segment was the longest, consisting of five tandem repeats that spanned 1659 bp with the highest A + T content of 72.2%. The circular map and genomic profile of the *A. moniliformis* mitogenome were summarized in Fig. 1 and Table 1, respectively. The entire mitogenome sequence was submitted to GenBank under the accession number PV138266.

**Table 1:** Mitochondrial genomic profile of *A. moniliformis* in the present study

Gene/region	Position	Strand	Size (bp)	No. of amino acids	Ini/ter codons^a^	Anti-codon	In^b^
tRNA-Ser^UCN^ (S_2_)	1–55	L	55			TGA	0
tRNA-Ile (I)	76–137	L	62			GAT	20
tRNA-Met (M)	140–201	L	62			CAT	2
*nad2*	202–1164	L	963	320	ATT/TAG		0
tRNA-Trp (W)	1163–1218	L	56			TCA	–2
tRNA-Cys (C)	1211–1263	H	53			GCA	–8
tRNA-Gln (Q)	1262–1327	H	66			TTG	–2
tRNA-Tyr (Y)	1330–1390	H	61			GTA	2
*cox1*	1392–2916	L	1525	508	CTG/T		1
tRNA-Leu^UUR^ (L_2_)	2917–2979	L	63			TAA	0
*cox2*	2980–3643	L	664	221	ATA/T		0
tRNA-Asp (D)	3644–3698	L	55			GTC	0
*atp8*	3699–3809	L	111	36	ATT/TAA		0
*atp6*	3806–4456	L	651	216	ATA/TAA		–4
*cox3*	4456–5236	L	781	260	ATG/T		–1
tRNA-Gly (G)	5237–5291	L	55			TCC	0
*nad3*	5292–5636	L	345	114	ATC/TAA		0
tRNA-Ala (A)	5635–5691	L	57			TGC	–2
tRNA-Arg (R)	5691–5751	L	61			TCG	–1
tRNA-Lys (K)	5751–5804	L	54			TTT	–1
tRNA-Asn (N)	5804–5858	L	55			GTT	–1
tRNA-Ser^AGN^ (S_1_)	5859–5913	L	55			TCT	0
tRNA-Glu (E)	5915–5969	L	55			TTC	1
tRNA-Phe (F)	5968–6024	H	57			GAA	–2
*nad5*	6025–7627	H	1603	534	ATG/T		0
tRNA-His (H)	7629–7689	H	61			GTG	1
*nad4*	7690–8950	H	1261	420	ATG/T		0
*nad4L*	8944–213	H	270	89	ATG/TAG		–7
tRNA-Thr (T)	9230–9284	L	55			TGT	16
tRNA-Pro (P)	9285–9340	H	56			TGG	0
*nad6*	9342–9785	L	444	147	ATC/TAA		1
*cytb*	9785–10,894	L	1110	369	ATG/TAA		–1
*nad1*	10,925–11,833	H	909	302	ATG/TAA		30
*rrnL*	11,834–12,977	H	1144				0
tRNA-Val (V)	12,978–13,034	H	57			TAC	0
*rrnS*	13,035–13,691	H	657				0
tRNA-Leu^CUN^ (L_1_)	13,692–13,751	H	60			TAG	0
NCR	13,752–16,367		2616				0

^a^Initiation and termination codons

^b^The length of the intergenic nucleotides

### Nucleotide composition and nucleotide diversity across six pentastomid mitogenomes

**Fig. 2:**
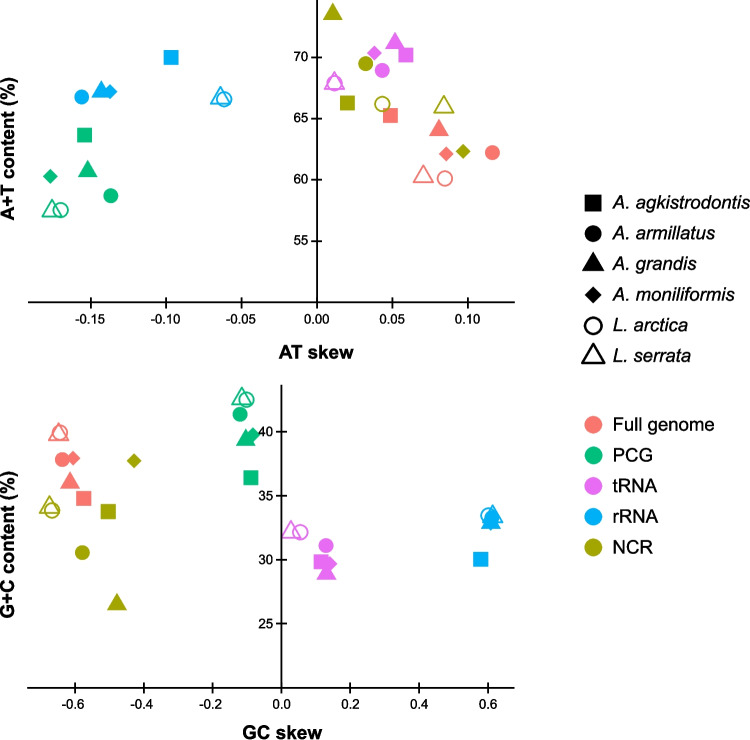
Relationships between nucleotide composition and skewness across mitochondrial genomes of six pentastomid species. Each species and its features are represented by a different symbol and color

The nucleotide composition and skewness of the 13 PCGs, two rRNA genes, 22 tRNA genes, and NCR were comparatively analyzed across *A. moniliformis* and other pentastomid mitogenomes (Table 2). The whole mitogenome of *A. moniliformis* exhibited a pronounced A + T content of 62.1%, slightly lower than that observed in other *Armillifer* spp. (62.2–65.2%), but higher than in *Linguatula* spp. (60.1–60.3%). All complete pentastomid mitogenomes displayed positive AT skew (from 0.049 to 0.116) and negative GC skew (− 0.648 to − 0.575) on the L-strand. Furthermore, the A + T content of the 13 PCGs located on both strands ranged from 57.5% (*Linguatula* spp.) to 63.6% (*A. agkistrodontis*), accompanied by negative AT skew (− 0.177 to − 0.137) and GC skew (− 0.120 to − 0.083). The A + T content of the 22 tRNA genes ranged from 67.9% (*Linguatula* spp.) to 71.1% (*A. grandis*). These tRNA genes exhibited positive AT skew (0.012 to 0.059) and GC skew (0.027 to 0.141). The A + T content of the *rrnS* and *rrnL* genes varied from 66.6% (*L. arctica*) to 70% (*A. agkistrodontis*), and both exhibited negative AT skew (− 0.156 to − 0.061) and positive GC skew (0.579 to 0.613). The A + T content of the NCRs ranged from 62.3% (*A. moniliformis*) to 73.5% (*A. grandis*). Based on the L-strand, all pentastomid NCRs exhibited positive AT skew (0.011 to 0.097) and negative GC skew − 0.674 to − 0.428) (Fig. 2). Sequence polymorphism of the longest repetitive NCR segments consisting of five tandem repeats across four *Armillifer* species was also demonstrated by entropy plot analysis (Fig. S2).

**Table 2:** Nucleotide composition and skewness of the complete mitogenomes of six pentastomid species

Species	Feature	Length (bp)	A (%)	C (%)	G (%)	T (%)	A + T %	AT skew	GC skew
*A. agkistrodontis*	Whole mitogenome	16,521	5652 (34.2)	4521 (27.4)	1221 (7.4)	5127 (31.0)	65.2	0.049	− 0.575
	PCGs	10,650	2867 (26.9)	2109 (19.8)	1764 (16.6)	3910 (36.7)	63.6	− 0.154	− 0.089
	tRNAs	1281	476 (37.2)	169 (13.2)	213 (16.6)	423 (33.0)	70.2	0.059	0.115
	rRNAs	1803	570 (31.6)	114 (6.3)	427 (23.7)	692 (38.4)	70.0	− 0.097	0.579
	NCR	2769	936 (33.8)	702 (25.4)	232 (8.4)	899 (32.5)	66.3	0.020	− 0.503
*A. armillatus*	Whole mitogenome	16,738	5812 (34.7)	5177 (30.9)	1149 (6.9)	4600 (27.5)	62.2	0.116	− 0.637
	PCGs	10,652	2698 (25.3)	2466 (23.2)	1936 (18.2)	3552 (33.4)	58.7	− 0.137	− 0.120
	tRNAs	1271	457 (36.0)	172 (13.5)	223 (17.6)	419 (33.0)	68.9	0.043	0.129
	rRNAs	1807	509 (28.2)	117 (6.5)	484 (26.8)	697 (38.6)	66.7	− 0.156	0.611
	NCR^1^	2984	1070 (35.9)	719 (24.1)	192 (6.4)	1003 (33.6)	69.5	0.032	− 0.578
*A. grandis*	Whole mitogenome	16,071	5562 (34.6)	4665 (29.0)	1115 (7.0)	4729 (29.4)	64.0	0.081	− 0.614
	PCGs	10,653	2741 (25.7)	2312 (21.7)	1877 (17.6)	3723 (35.0)	60.7	− 0.152	− 0.104
	tRNAs	1275	477 (37.4)	160 (12.6)	208 (16.3)	430 (33.7)	71.1	0.052	0.130
	rRNAs	1803	519 (28.8)	116 (6.4)	476 (26.4)	692 (38.4)	67.2	− 0.143	0.608
	NCR^2^	2315	860 (37.2)	453 (19.6)	160 (6.9)	842 (36.4)	73.5	0.011	− 0.478
*A. moniliformis*	Whole mitogenome	16,367	5517 (33.7)	4980 (30.4)	1224 (7.5)	4646 (28.4)	62.1	0.086	− 0.605
	PCGs	10,637	2639 (24.8)	2289 (21.5)	1937 (18.2)	3772 (35.5)	60.3	− 0.177	− 0.083
	tRNAs	1271	464 (36.5)	162 (12.8)	215 (16.9)	430 (33.8)	70.3	0.038	0.141
	rRNAs	1801	522 (29.0)	116 (6.4)	475 (26.4)	688 (38.2)	67.2	− 0.137	0.607
	NCR	2616	894 (34.2)	704 (26.9)	282 (10.8)	736 (28.1)	62.3	0.097	− 0.428
*L. arctica*	Whole mitogenome^3^	14,787	4820 (32.6)	4851 (32.8)	1050 (7.1)	4066 (27.5)	60.1	0.085	− 0.644
	PCGs	10,690	2552 (23.9)	2502 (23.4)	2039 (19.1)	3597 (33.7)	57.5	− 0.170	− 0.102
	tRNAs	1264	434 (34.3)	192 (15.2)	214 (16.9)	424 (33.5)	67.9	0.012	0.054
	rRNAs	1783	557 (31.2)	119 (6.7)	477 (26.8)	630 (35.3)	66.6	− 0.061	0.601
	NCR	1011	349 (34.5)	285 (28.2)	57 (5.6)	320 (31.7)	66.2	0.043	− 0.667
*L. serrata*	Whole mitogenome	15,328	4943 (32.3)	5020 (32.8)	1073 (7)	4292 (28.0)	60.3	0.070	− 0.648
	PCGs	10,624	2516 (23.7)	2521 (23.7)	1999 (18.8)	3588 (33.8)	57.5	− 0.176	− 0.115
	tRNAs	1273	437 (34.3)	199 (15.6)	210 (16.5)	427 (33.5)	67.9	0.012	0.027
	rRNAs	1782	556 (31.2)	115 (6.5)	479 (26.9)	632 (35.5)	66.7	− 0.064	0.613
	NCR	1550	554 (35.7)	442 (28.5)	86 (5.6)	468 (30.2)	65.9	0.084	− 0.674

^1^Nine base positions (9Ns) are present in *A. armillatus* mitochondrial NCR

^2^Two base positions (Y and R) are present in *A. grandis* mitochondrial NCR

^3^Two base positions (2Ns) are present in *L. arctica* mitochondrial intergenic region

**Fig. 3:**
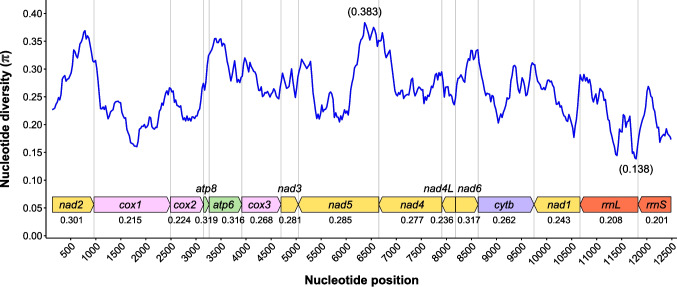
Sliding window analysis of 13 PCGs and two rRNA genes across six pentastomid mitogenomes. The average nucleotide diversity value of each gene was indicated beneath the gene name. The blue line was plotted with a sliding window of 250 bp with a step size of 25 bp

Nucleotide diversity (*π*) of the 13 PCGs and two rRNA genes across six pentastomid mitogenomes was demonstrated by sliding window analysis (Fig. 3). The most variable region (positions 6241–6496) was located in *nad5* (*π* = 0.383), while the most conserved fragment (11,663−11,913) was in *rrnL* (*π* = 0.138). The most conserved PCGs were *cox1* (*π* = 0.215) and *cox2* (*π* = 0.224), while *atp6*, *atp8*, and *nad6* were highly variable with *π* values of 0.316, 0.319, and 0.317, respectively. Two rRNA genes were highly conserved, with *π* values of 0.208 in *rrnL* and 0.201 in *rrnS*.

### Codon usage and RSCU analyses

**Fig. 4:**
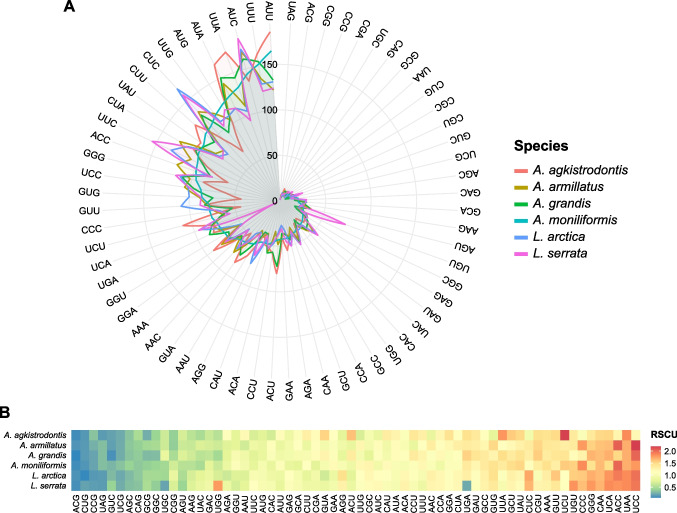
**A** Codon usage plot across six pentastomid species. Codons are arranged clockwise by an increasing frequency found in *A. moniliformis* (teal line). Each spike was plotted using the polar coordinate system, representing the frequency of each codon. **B** The heat map of RSCU values was calculated for each codon across six pentastomid species. RSCU values are illustrated by the color spectrum on the right

Codon usage and RSCU in *A. moniliformis* were analyzed (Table 3) and compared to those of the other five pentastomid mitogenomes. The frequency pattern of codon usage is similar between *A. moniliformis* and the other pentastomids (Fig. 4A). For *A. moniliformis*, the start codon of most genes was ATN, with ATG being the most common, whereas *cox1* started with CTG. Most genes used TAA and TAG as stop codons, with TAA being the most common for six PCGs (*atp6*, *atp8*, *cytb*, *nad1*, *nad3*, and *nad6*) and TAG for two PCGs (*nad2* and *nad4L*). The remaining genes (*cox1*, *cox2*, *cox3*, *nad4*, and *nad5*) used an incomplete stop codon consisting of a single T nucleotide that was post-transcriptionally modified to form a complete stop codon with an additional two 3′ A nucleotides (TAA) through polyadenylation.

**Table 3:** Codon usage analysis of 13 mitochondrial PCGs in *A. moniliformis*

Amino acid	Codon	No. of usage	Percent	RSCU	Amino acid	Codon	No. of usage	Percent	RSCU
Ala (A)	GCG	10	0.28%	0.388	Pro (P)	CCG	8	0.23%	0.189
Ala (A)	GCA	20	0.56%	0.777	Pro (P)	CCA	38	1.07%	0.899
Ala (A)	GCU	38	1.07%	1.476	Pro (P)	CCU	45	1.27%	1.065
Ala (A)	GCC	35	0.99%	1.359	Pro (P)	CCC	78	2.20%	1.846
Cys (C)	UGU	27	0.76%	1.543	Gln (Q)	CAG	9	0.25%	0.360
Cys (C)	UGC	8	0.23%	0.457	Gln (Q)	CAA	41	1.16%	1.640
Asp (D)	GAU	31	0.87%	1.216	Arg (R)	CGG	7	0.20%	0.667
Asp (D)	GAC	20	0.56%	0.784	Arg (R)	CGA	8	0.23%	0.762
Glu (E)	GAG	29	0.82%	0.817	Arg (R)	CGU	14	0.39%	1.333
Glu (E)	GAA	42	1.18%	1.183	Arg (R)	CGC	13	0.37%	1.238
Phe (F)	UUU	152	4.28%	1.206	Ser1 (S_1_)	AGG	50	1.41%	1.018
Phe (F)	UUC	100	2.82%	0.794	Ser1 (S_1_)	AGA	42	1.18%	0.855
Gly (G)	GGG	95	2.68%	1.445	Ser1 (S_1_)	AGU	27	0.76%	0.550
Gly (G)	GGA	70	1.97%	1.065	Ser1 (S_1_)	AGC	20	0.56%	0.407
Gly (G)	GGU	70	1.97%	1.065	Ser2 (S_2_)	UCG	16	0.45%	0.326
Gly (G)	GGC	28	0.79%	0.426	Ser2 (S_2_)	UCA	73	2.06%	1.486
His (H)	CAU	49	1.38%	1.181	Ser2 (S_2_)	UCU	77	2.17%	1.567
His (H)	CAC	34	0.96%	0.819	Ser2 (S_2_)	UCC	88	2.48%	1.791
Ile (I)	AUU	165	4.65%	1.068	Thr (T)	ACG	4	0.11%	0.082
Ile (I)	AUC	144	4.06%	0.932	Thr (T)	ACA	49	1.38%	1.010
Lys (K)	AAG	23	0.65%	0.541	Thr (T)	ACU	43	1.21%	0.887
Lys (K)	AAA	62	1.75%	1.459	Thr (T)	ACC	98	2.76%	2.021
Leu2 (L_2_)	UUG	118	3.32%	1.202	Val (V)	GUG	86	2.42%	1.445
Leu2 (L_2_)	UUA	133	3.75%	1.355	Val (V)	GUA	58	1.63%	0.975
Leu1 (L_1_)	CUG	12	0.34%	0.122	Val (V)	GUU	78	2.20%	1.311
Leu1 (L_1_)	CUA	104	2.93%	1.059	Val (V)	GUC	16	0.45%	0.269
Leu1 (L_1_)	CUU	110	3.10%	1.121	Trp (W)	UGG	34	0.96%	0.642
Leu1 (L_1_)	CUC	112	3.16%	1.141	Trp (W)	UGA	72	2.03%	1.358
Met (M)	AUG	123	3.47%	0.980	Tyr (Y)	UAU	104	2.93%	1.518
Met (M)	AUA	128	3.61%	1.020	Tyr (Y)	UAC	33	0.93%	0.482
Asn (N)	AAU	55	1.55%	0.957	Stop	UAG	2	0.06%	0.308
Asn (N)	AAC	60	1.69%	1.043	Stop	UAA	11	0.31%	1.692

**Fig. 5:**
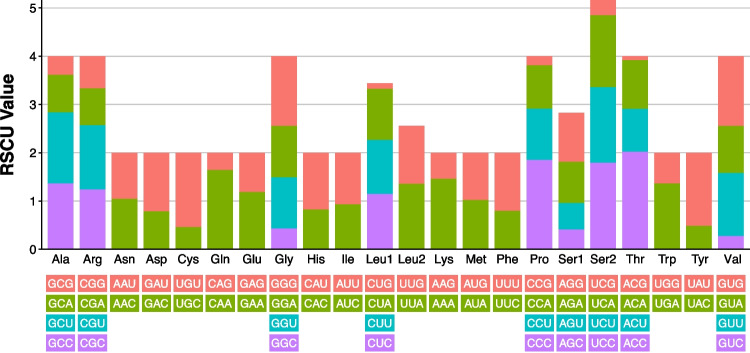
Relative synonymous codon usage (RSCU) values calculated for each codon across 13 PCGs of the *A. moniliformis* mitogenome. The stop codon was not given

For *A. moniliformis*, four codons, ACC (T), CCC (P), UCC (S_2_), and CAA (Q), were overrepresented with RSCU values greater than 1.6. Conversely, 13 codons, ACG (T), CUG (L_1_), CCG (P), GUC (V), UCG (S_2_), CAG (Q), GCG (A), AGC (S_1_), GGC (G), UGC (C), UAC (Y), AAG (K), and AGU (S_1_), were underrepresented (RSCU value < 0.6), with most ending in G or C. It was also observed that A or T frequently appeared in the third position of degenerate codons (Fig. 5). The RSCU values of *A. moniliformis* and other pentastomid species were calculated and summarized in Fig. 4B.

Two codon families, which code for Leu1 (L_1_) and Ile (I), were used more predominantly than those of other amino acids in *A. moniliformis* (Table 3). These branched-chain hydrophobic amino acids served as the major components of several transmembrane proteins responsible for energy production, which was the pivotal function of the mitochondria. The codon family (TGT/TGC) coding for Cys (C) was the least used.

### Phylogenetic analysis

**Fig. 6:**
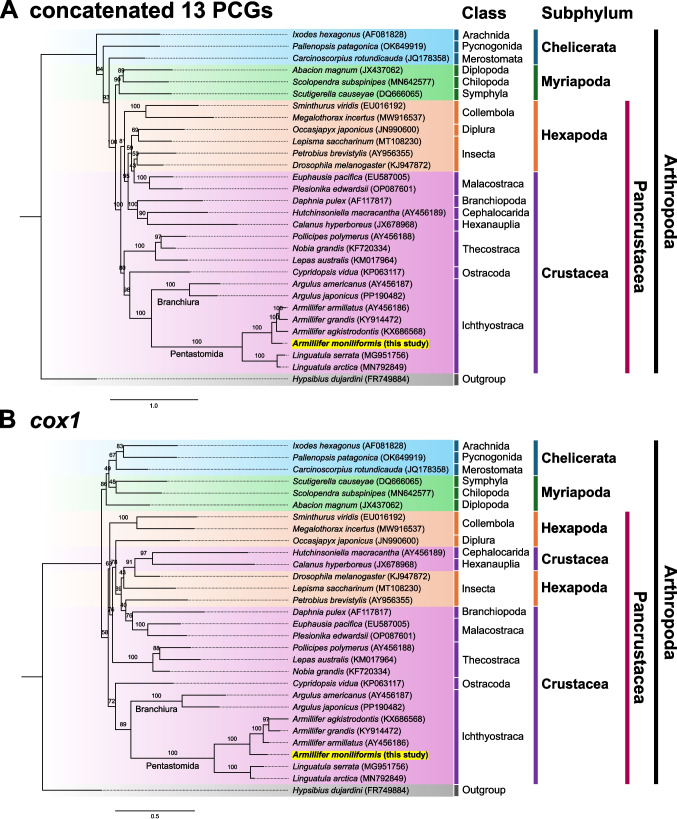
Phylogenetic relationships of *A. moniliformis*, other pentastomids, and other arthropod groups based on sequences of the concatenated 13 PCGs (**A**) and the *cox1* gene (**B**) using the maximum likelihood method. *Hypsibius dujardini* was used as an outgroup

The ML phylogenetic trees were constructed using the single gene (*cox1*) and full concatenation approaches, using the concatenated nucleic acid sequences of 13 PCGs, both obtained from *A. moniliformis* and 28 other arthropods, with *H. dujardini* as the outgroup. The concatenation approach used the best-performing substitution model for each partition (Table S5). Based on both phylogenies, it was clear that our complete mitochondrial sequence of *A. moniliformis* identified in this study clustered with those of *Armillifer* spp. with high bootstrap support, as depicted in Fig. 6. In addition, the *Armillifer* sequences exhibited a close relationship with those of *Linguatula*, both belonging to the subclass Pentastomida. The Pentastomida clade shared a common ancestor with *Argulus* spp. (fish lice) in the Branchiura clade and *Cypridopsis vidua* (seed shrimp) in the Ostracoda clade, forming the superclass Oligostraca. Based on the concatenation approach, the Pancrustacea clade, consisting of Crustacea and Hexapoda subphyla, was monophyletic and descended from a shared ancestor. A congruent pattern is shown by the *cox1* tree, which also exhibits a monophyletic configuration of the Pancrustacea clade.

## Discussion

It has been known that four species of *Armillifer*, namely *A. armillatus*, *A. agkistrodontis*, *A. grandis*, and *A. moniliformis*, can cause pentastomiasis in humans (Tappe and Warrell 2020). Cases of pentastomiasis were primarily diagnosed based on surgical or autopsy findings of parasitic larvae or radiological evidence of calcified larvae. It is noteworthy that *Armillifer* species exhibit significant morphological similarity, particularly during the immature stage (Tappe & Buttner 2009). However, all previously reported cases in Southeast Asia were presumptively diagnosed as *A. moniliformis* due to its documented geographical distribution. Therefore, there is an urgent need to develop precise diagnostic methodologies to identify these parasites accurately.

As previously mentioned, molecular diagnostics will facilitate the confirmation of species identification. Mitochondrial markers have been utilized more extensively than their nuclear counterparts for identifying parasite species, including pentastomids (Tappe et al. 2016). The mitochondrial genomes of several *Armillifer* species have previously been characterized, including *A. armillatus* (Lavrov et al. 2004; Lemarcis et al. 2020), *A. agkistrodontis* (Li et al. 2016), and *A. grandis* (Grau et al. 2017). However, the mitochondrial genome of *A. moniliformis* has not been investigated. Therefore, this study is the first to characterize the complete mitogenome of *A. moniliformis*, using nanopore long-read sequencing. The key findings of this study will provide deeper insights into pentastomid biology and significantly enhance the development of molecular diagnostics, overcoming the limitations of traditional methods.

Our comparative analysis revealed high levels of similarity in terms of gene content, size, organization, nucleotide composition, and codon usage between the mitogenomes of *A. moniliformis* in this study and those of other previously sequenced *Armillifer* and *Linguatula* species. The nucleotide composition of the complete mitogenomes of these species showed a clear overrepresentation of A and T, with A + T content exceeding 60%, and A slightly higher than T. Consistently, the skew statistics of all pentastomid mitogenomes indicated strand asymmetry, with positive AT skew and negative GC skew on the L-strand. This strand asymmetry is universally observed in metazoan mitochondrial genomes, primarily due to the asymmetric replication process of mitochondrial DNA and biased mutation patterns (Wei et al. 2010; Zhang et al. 2015). During mitochondrial DNA replication, one strand remains single-stranded for longer, making it more susceptible to the spontaneous deamination of adenine and cytosine. This results in strand-specific mutations and compositional bias between the two mitochondrial strands.

However, the length of the entire mitogenome varied across *Linguatula* and *Armillifer* species, primarily due to the length of the NCR, which ranges from 1011 to 1550 bp in *Linguatula* spp. and 2315 to 2984 bp in *Armillifer* spp. Interestingly, this variation in the NCR length is due to differences in repeat motif sequences and their copy number between species. In *A. moniliformis*, three repetitive segments were identified, each differing in period size and A + T content. The third NCR segment is the longest at 1659 bp and consists of five tandem repeats with the highest A + T content of 72.2%. A similar high A + T bias was also observed in the longest repetitive NCR segment in *A. armillatus*, *A. grandis*, and *A. agkistrodontis*. This suggests that this tandemly repeated sequence likely facilitates the initiation of mitochondrial DNA replication in this genus by acting as binding sites for the replication complex or by forming secondary structures necessary for the origin of replication to function (Chen et al. 2011; Cook 2005). Despite this segment exhibiting a similar high A + T bias across species, significant genetic distances of 21.4–40.6% were observed among *Armillifer* species (Table S6, Fig. S2), indicating its potential as a marker for distinguishing pentastomid species.

Utilizing a sliding window approach, *cox1* was identified as the most conserved PCG, with the two rRNA genes (*rrnS* and *rrnL*) also being highly conserved. Conversely, *atp6*, *atp8*, and *nad6* exhibited greater sequence variability across species. These results suggest that *atp6*, *atp8*, and *nad6* may be more suitable markers for species identification and population genetic analysis than the less variable *cox1*, which is conventionally employed as a DNA barcode for taxonomic classification and phylogenetic inference (Hebert et al. 2003).

For this study, we selected 28 complete mitochondrial genomes from GenBank for phylogenetic analysis. Although the database contains numerous arthropod mitochondrial genomes, we chose these 28 because they have a full set of the 13 annotated PCGs, representing each class within the phylum Arthropoda. According to our phylogenetic results based on single-gene and concatenation approaches, *A. moniliformis* exhibited a monophyletic relationship with other *Armillifer* spp., forming a clade distinct from *Linguatula* pentastomids, which have canids and felids as definitive hosts (Shamsi et al. 2022). Our phylogenetic trees also demonstrated that the monophyletic subclass Pentastomida and the subclass Branchiura are sister groups, with both taxa belonging to the class Ichthyostraca. It is noteworthy that close proximities of two African lineages (*A. armillatus* and *A. grandis*) as well as of two oriental lineages (*A. agkistrodontis* and *A. moniliformis*) could only be observed in the concatenated tree, indicating phylogeographic relationships across these species. In addition, the placement of *A. moniliformis* within a monophyletic pentastomid clade in our phylogenetic trees was congruent with findings from previous studies that used the 18S rRNA gene (Li et al. 2012, 2024; Rajapaksha et al. 2020).

However, there are some discrepancies between these two approaches. Specifically, certain Crustacea lineages are found intermingled with Hexapoda lineages in the *cox1* tree. In contrast, the concatenation-based tree places each lineage according to its taxonomic classification. This suggests that the topology of the latter is more accurate. This observation indicates that the utility of the *cox1* phylogenetic approach may be somewhat limited in terms of its performance and informativeness for a given taxon, compared to the full concatenation approach. Furthermore, the topology of single gene-based phylogenies using genes other than *cox1,* such as *nad4* and *nad5*, also differs due to variations in evolutionary rate, sequence length, nucleotide composition, and transition/transversion mutation bias between genes (Fig. S3) (Gadagkar et al. 2005). Consequently, mitogenome phylogenetics is more precise than single-gene analysis because it integrates comprehensive data from multiple genes with an optimized substitution model for each PCG. This improves phylogenetic resolution across a wide range of divergence depths and reduces bias arising from variability between genes. Therefore, the full concatenation approach can resolve controversies arising from different tree topologies generated from different single genes.

In Thailand, ten cases of human pentastomiasis have previously been formally documented, predominantly in the northern and northeastern provinces (Manunpichu 1981; Sambhavaphol and Orparyoon 1985; Sarasas et al. 1976; Sirisumpan et al. 1968; Tescharoen and Laohapand 1986; Thamprasert 1992; Tiewchaloren et al. 1999). Most cases were diagnosed as *A. moniliformis* infections, and two cases exhibited a history of consuming undercooked snake meat (Sarasas et al. 1976; Thamprasert 1992). The most recent case from which we obtained larval specimens also exhibited a history of consuming local food prepared from snake meat. To the best of our knowledge, *A. moniliformis* has been identified in the feces and carcasses of several snake species in Southeast Asia, India, and Sri Lanka, including Malaysian blood python (*Python curtus*) (Tongtako et al. 2013), Indian python (*P. molurus)* (Riley and Self 1981), reticulated python (*P. reticulatus*) (Krishnasamy et al. 1981), Burmese python (*P. bivittatus*) (Krishnasamy et al. 1981), and common rat snake (*Ptyas mucosus*) (Fernando and Fernando 2014). Additionally, pentastomes of an as yet undetermined species were identified in Siamese cobras (*Naja kaouthia*) and a king cobra (Dubey et al. 2017; Vasaruchapong et al. 2017). Consequently, several snake species can potentially serve as sources of zoonotic infection through the consumption of traditional snake meat dishes, as previously mentioned.

Apart from snakes, all pentastomid species require mammalian intermediate hosts to complete their transmission cycle. *Armillifer moniliformis* has been identified in several mammalian hosts in Sri Lanka and Malaysia, including the brown palm civet (*Paradoxurus montanus*) (Rajapaksha et al. 2020), binturong (*Arctictis binturong*) (Stabler and Self 1967), small Indian civet (*Viverricula indica mayori*) (Paramananthan 1962), Indian wild boar (*Sus cristatus cristatus*) (Paramananthan 1962), and Malaysian long-tailed macaque (*Macaca irus*) (Burns-Cox et al. 1969). Recently, *A*. *moniliformis* was also reported in a cynomolgus macaque (*Macaca fascicularis*) at a laboratory animal center in China (Li et al. 2024). This macaque was purchased from a company that frequently imports from Laos, where the infection may have occurred. In Thailand, visceral pentastomiasis caused by *A. moniliformis* was diagnosed in a domestic dog in Bangkok, the capital city, whereas that caused by *A. armillatus* was diagnosed in a captive striped hyena (*Hyaena hyaena*) in Chiang Mai Province, which was imported from Tanzania in East Africa (Dechkajorn et al. 2016). Furthermore, *A. armillatus* was detected in an African lorisid (*Perodicticus potto*), a nocturnal non-human primate, in the Republic of the Congo (Lemarcis et al. 2020). This information suggests the possibility of *A. armillatus* spillover into Thailand through the importation of exotic animal species from Africa, given its known endemicity in this region.

As aforementioned, it is crucial to state that *Armillifer* pentastomids can exploit a wide variety of definitive and intermediate hosts, including humans, indicating the complexity of zoonotic transmission. However, the significant epidemiological information, such as the diversity of pentastomid species, infection prevalence in animals and humans, and associated risk factors, remains underinvestigated. Conventionally, the diagnosis was performed solely based on morphological microscopic examination, which might not accurately determine the parasite species and limit further epidemiological studies. Therefore, an alternative diagnostic tool that can identify parasite species with high sensitivity and accuracy is necessary. Ultimately, the novel mitogenome sequence information of *A. moniliformis* generated from this study will be implemented as raw key data for the development of molecular diagnostics. This will be beneficial for clinical diagnosis and epidemiological surveillance of pentastomiasis, leading to more effective prevention and control of this neglected zoonotic disease.

## Conclusions

This research unveiled the first complete mitochondrial genome of *A. moniliformis* widespread in Southeast Asia. Comparative sequence and phylogenetic analyses revealed close genetic similarity of *A. moniliformis* to other *Armillifer* species and supported the placement of all members of the subclass Pentastomida in the subphylum Crustacea. The novel advanced knowledge from this study not only offers new molecular markers for species identification, diagnostic development, and epidemiological study but also provides deeper insights into the evolutionary biology of *Armillifer* pentastomids, neglected parasites of zoonotic importance.

## Supplementary Information

Below is the link to the electronic supplementary material.

Supplementary file1 (DOCX 29 KB)

Supplementary file2 (DOCX 29 KB)

Supplementary file3 (DOCX 32 KB)

Supplementary file4 (DOCX 607 KB)

Supplementary file5 (DOCX 28.1 KB)

Supplementary file6 (DOCX 27 KB)

Supplementary file7 (DOCX 27.6 KB)

Supplementary file8 (DOCX 896 KB)

Supplementary file9 (DOCX 537 KB)

## Data Availability

The *cox1*, *nad5*, ITS2, 18S rRNA, and 28S rRNA sequences of *A. moniliformis* were deposited in the GenBank database under accession numbers PP272417, PP273415, PP273236, PV124096, and PV132650, respectively. The mitochondrial genome sequence of *A. moniliformis* has also been deposited in the GenBank database under the accession number PV138266.

## Abbreviations

18S rRNA: Small subunit ribosomal RNA
28S rRNA: Large subunit ribosomal RNA
*atp6*: ATP synthase F0 subunit 6
*atp8*: ATP synthase F0 subunit 8
bp: Base pair
*cox1*: Cytochrome c oxidase subunit I
*cox2*: Cytochrome c oxidase subunit II
*cox3*: Cytochrome c oxidase subunit III
Cys: Cysteine
*cytb*: Cytochrome b
DNA: Deoxyribonucleic acid
gDNA: Genomic DNA
H-strand: Heavy strand
Ile: Isoleucine
IPTG: Isopropyl‑beta‑D‑1‑thiogalactopyranoside
ITS2: Internal transcribed spacer 2
L-strand: Light strand
LB: Luria-Bertani
Leu: Leucine
Mb: Megabase
ML: Maximum likelihood
*nad1*: NADH dehydrogenase subunit 1
*nad2*: NADH dehydrogenase subunit 2
*nad3*: NADH dehydrogenase subunit 3
*nad4*: NADH dehydrogenase subunit 4
*nad4L*: NADH dehydrogenase subunit 4L
*nad5*: NADH dehydrogenase subunit 5
*nad6*: NADH dehydrogenase subunit 6
NCR: Non-coding region
PCG: Protein-coding gene
PCR: Polymerase chain reaction
RNA: Ribonucleic acid
rRNA: Ribosomal RNA
*rrnL*: Large subunit (16S) ribosomal RNA
*rrnS*: Small subunit (12S) ribosomal RNA
RSCU: Relative synonymous codon usage
tRNA: Transfer RNA
X‑Gal: 5‑Bromo‑4‑chloro‑3‑indolyl‑beta‑D‑galactoside
*π*: Nucleotide diversity
