# Supplementary material for: First complete mitochondrial genome of Armillifer moniliformis (Pentastomida: Porocephalida) isolated from a human case in Northern Thailand: comparative and phylogenetic analyses
Source: Parasitol Res. 2025 Jun 27;124(6):69. doi: 10.1007/s00436-025-08516-x (PMC12202648; doi:10.1007/s00436-025-08516-x)
Supplement: Supplementary file 1 — Supplementary file1 (DOCX 29 KB) [file 436_2025_8516_MOESM1_ESM.docx]

**Table S1** Oligonucleotide primers for pentastomid species identification

| Gene region | Primer name | Sequence (5´ → 3´) | Annealing temperature (°C) | Reference |
| --- | --- | --- | --- | --- |
| Cytchrome c oxidase subunit I (*cox1*) | F: LCO-1490 | GGTCAACAAATCATAAAGATATTGG | 55 | Folmer et al. 1994 |
|  | R: HCO-2198 | TAAACTTCAGGGTGACCAAAAAATC |  |  |
| Internal transcribed spacer 2 (ITS2) | F: 3S | CGGTGGATCACTCGGCTCGT | 55 | Bowles et al. 1995 |
|  | R: 28A | CCTGGTTAGTTTCTTTTCCTCCGC |  |  |
| NADH dehydrogenase subunit 5 (*nad5*) | F: ASL forward | TTACTCCAACCAAAGGTATA | 55 | Rajapaksha et al. 2020 |
|  | R: ASL reverse | TCGTCGACTCTTGTGACCTC |  |  |
| Small subunit ribosomal RNA (18S rRNA) | F: 18S rRNA-F | AACCTGGTTGATCCTGCCAGTAG | 58 | Medlin et al. 1988 |
|  | R: 18S rRNA-R | GATCCTTCTGCAGGTTCACCTAC |  |  |
| Large subunit ribosomal RNA (28S rRNA) | F: LSU5 | ACCCGCTGAATTTAAGCA | 58 | Littlewood and Johnston 1995 |
|  | R: LSU3 | TCCTGAGGGAAACTTCGG |  |  |

**References**

1. Folmer O, Black M, Hoeh W, Lutz R, Vrijenhoek R (1994) DNA primers for amplification of mitochondrial cytochrome c oxidase subunit I from diverse metazoan invertebrates. Mol Mar Biol Biotechnol 3:294-299

2. Bowles J, Blair D, McManus DP (1995) A molecular phylogeny of the human schistosomes. Mol Phylogenet Evol 4:103-109. https://doi.org/10.1006/mpev.1995.1011

3. Rajapaksha C, Amarasinghe AP, Fernando S, Rajapakse R, Tappe D, Wickramasinghe S (2020) Morphological and molecular description of *Armillifer moniliformis* larvae isolated from Sri Lankan brown palm civet (*Paradoxurus montanus*). Parasitol Res 119:773-781. https://doi.org/10.1007/s00436-019-06581-7

4. Medlin L, Elwood HJ, Stickel S, Sogin ML (1988) The characterization of enzymatically amplified eukaryotic 16S-like rRNA-coding regions. Gene 71:491-499. https://doi.org/10.1016/0378-1119(88)90066-2

5. Littlewood DTJ, Johnston DA (1995) Molecular phylogenetics of the four *Schistosoma* species groups determined with partial 28S ribosomal RNA gene sequences. Parasitology. 111:167-175. https://doi.org/10.1017/s003118200006491x
