# Supplementary material for: First complete mitochondrial genome of Armillifer moniliformis (Pentastomida: Porocephalida) isolated from a human case in Northern Thailand: comparative and phylogenetic analyses
Source: Parasitol Res. 2025 Jun 27;124(6):69. doi: 10.1007/s00436-025-08516-x (PMC12202648; doi:10.1007/s00436-025-08516-x)
Supplement: Supplementary file 2 — Supplementary file2 (DOCX 29 KB) [file 436_2025_8516_MOESM2_ESM.docx]

**Table S2** Sanger sequencing primers for the validation of nanopore sequence assembly

| Region | Sequence (5´ → 3´) | Base position | T_a_ (°C) | GC% | Amplicon size (bp) |
| --- | --- | --- | --- | --- | --- |
| Cytochrome c oxidase subunit I (*cox1*) | F: TAAGGCACTGCCCCTTCCTACG | 1269-1290 | 55 | 59.1 | 1763 |
|  | R: GTTCTATAATGGGGGAAGCTG | 3031-3011 |  | 47.6 |  |
| Cytochrome c oxidase subunit II (*cox2*) | F: GAATGATTAAACAATATTCCCCCATCC | 2862-2888 | 56 | 37.0 | 863 |
|  | R: CAATGGAGGGGTATCATGTGAGG | 3724-3702 |  | 52.2 |  |
| NADH dehydrogenase subunit 5 (*nad5*) | F: CATTATATTCCTCATTTCTTTAGTG | 5902-5926 | 50 | 28.0 | 1838 |
|  | R: GATACATAGATTTTGATTTGTAAGGG | 7739-7714 |  | 30.8 |  |
| Cytochrome b (*cytb*) | F: CTATCTCCCCCTACCTATCCTCCTC | 9677-9701 | 60 | 56.0 | 1294 |
|  | R: GTGGGGGTATGTTGTTGCTGTGG | 10970-10948 |  | 56.5 |  |
| Large subunit (16S) ribosomal RNA (*rrnL*) | F: TCAACCAATGTCACAAAAGCCAG | 11757-11779 | 55 | 43.5 | 1114 |
|  | R: TAGGAGGATTTAAGGGTTTGTACC | 12870-12847 |  | 41.7 |  |
| Non-coding region (NCR) | F: TAAAATTCCTGCTAAAAGCCTTAGC | 13683-13707 | 56 | 36.0 | 2809 |
|  | R: GATAGCATATATTCCGTATCGAGG | 124-101 |  | 41.7 |  |

T_a_: Annealing temperature
