# Supplementary material for: First complete mitochondrial genome of Armillifer moniliformis (Pentastomida: Porocephalida) isolated from a human case in Northern Thailand: comparative and phylogenetic analyses
Source: Parasitol Res. 2025 Jun 27;124(6):69. doi: 10.1007/s00436-025-08516-x (PMC12202648; doi:10.1007/s00436-025-08516-x)
Supplement: Supplementary file 3 — Supplementary file3 (DOCX 32 KB) [file 436_2025_8516_MOESM3_ESM.docx]

**Table S3** List of mitochondrial genomes used for phylogenetic analysis in this study

| Phylum | Subphylum | Class | Subclass | Order | Species | Accession number |
| --- | --- | --- | --- | --- | --- | --- |
| Arthropoda | Chelicerata | Arachnida | Acari | Ixodida | *Ixodes hexagonus* | AF081828 |
| Arthropoda | Chelicerata | Merostomata | - | Xiphosura | *Carcinoscorpius rotundicauda* | JQ178358 |
| Arthropoda | Chelicerata | Pycnogonida | - | Pantopoda | *Pallenopsis patagonica* | OK649919 |
| Arthropoda | Crustacea | Branchiopoda | Phyllopoda | Diplostraca | *Daphnia pulex* | AF117817 |
| Arthropoda | Crustacea | Cephalocarida | Cephalocarida | Brachypoda | *Hutchinsoniella macracantha* | AY456189 |
| Arthropoda | Crustacea | Hexanauplia | Copepoda | Calanoida | *Calanus hyperboreus* | JX678968 |
| Arthropoda | Crustacea | Ichthyostraca | Branchiura | Arguloida | *Argulus americanus* | AY456187 |
| Arthropoda | Crustacea | Ichthyostraca | Branchiura | Arguloida | *Argulus japonicus* | PP190482 |
| Arthropoda | Crustacea | Ichthyostraca | Pentastomida | Porocephalida | ***Armillifer moniliformis* (this study)** | **PV138266** |
| Arthropoda | Crustacea | Ichthyostraca | Pentastomida | Porocephalida | *Armillifer agkistrodontis* | KX686568 |
| Arthropoda | Crustacea | Ichthyostraca | Pentastomida | Porocephalida | *Armillifer armillatus* | AY456186 |
| Arthropoda | Crustacea | Ichthyostraca | Pentastomida | Porocephalida | *Armillifer grandis* | KY914472 |
| Arthropoda | Crustacea | Ichthyostraca | Pentastomida | Porocephalida | *Linguatula arctica* | MN792849 |
| Arthropoda | Crustacea | Ichthyostraca | Pentastomida | Porocephalida | *Linguatula serrata* | MG951756 |
| Arthropoda | Crustacea | Malacostraca | Eumalacostraca | Decapoda | *Plesionika edwardsii* | OP087601 |
| Arthropoda | Crustacea | Malacostraca | Eumalacostraca | Eucarida | *Euphausia pacifica* | EU587005 |
| Arthropoda | Crustacea | Ostracoda | Podocopa | Podocopida | *Cypridopsis vidua* | KP063117 |
| Arthropoda | Crustacea | Thecostraca | Cirripedia | - | *Lepas australis* | KM017964 |
| Arthropoda | Crustacea | Thecostraca | Cirripedia | Balanomorpha | *Nobia grandis* | KF720334 |
| Arthropoda | Crustacea | Thecostraca | Cirripedia | Pollicipedomorpha | *Pollicipes polymerus* | AY456188 |
| Arthropoda | Hexapoda | Collembola | - | Neelipleona | *Megalothorax incertus* | MW916537 |
| Arthropoda | Hexapoda | Collembola | - | Symphypleona | *Sminthurus viridis* | EU016192 |
| Arthropoda | Hexapoda | Diplura | - | Diplura | *Occasjapyx japonicus* | JN990600 |
| Arthropoda | Hexapoda | Insecta | - | Zygentoma | *Lepisma saccharinum* | MT108230 |
| Arthropoda | Hexapoda | Insecta | Monocondylia | Archaeognatha | *Petrobius brevistylis* | AY956355 |
| Arthropoda | Hexapoda | Insecta | Pterygota | Diptera | *Drosophila melanogaster* | KJ947872 |
| Arthropoda | Myriapoda | Chilopoda | Pleurostigmophora | Scolopendromorpha | *Scolopendra subspinipes* | MN642577 |
| Arthropoda | Myriapoda | Diplopoda | Helminthomorpha | Callipodida | *Abacion magnum* | JX437062 |
| Arthropoda | Myriapoda | Symphyla | - | - | *Scutigerella causeyae* | DQ666065 |
| Tardigrada | - | Eutardigrada | - | Parachela | *Hypsibius dujardini* | FR749884 |
