# Supplementary material for: First complete mitochondrial genome of Armillifer moniliformis (Pentastomida: Porocephalida) isolated from a human case in Northern Thailand: comparative and phylogenetic analyses
Source: Parasitol Res. 2025 Jun 27;124(6):69. doi: 10.1007/s00436-025-08516-x (PMC12202648; doi:10.1007/s00436-025-08516-x)
Supplement: Supplementary file 4 — Supplementary file4 (DOCX 607 KB) [file 436_2025_8516_MOESM4_ESM.docx]

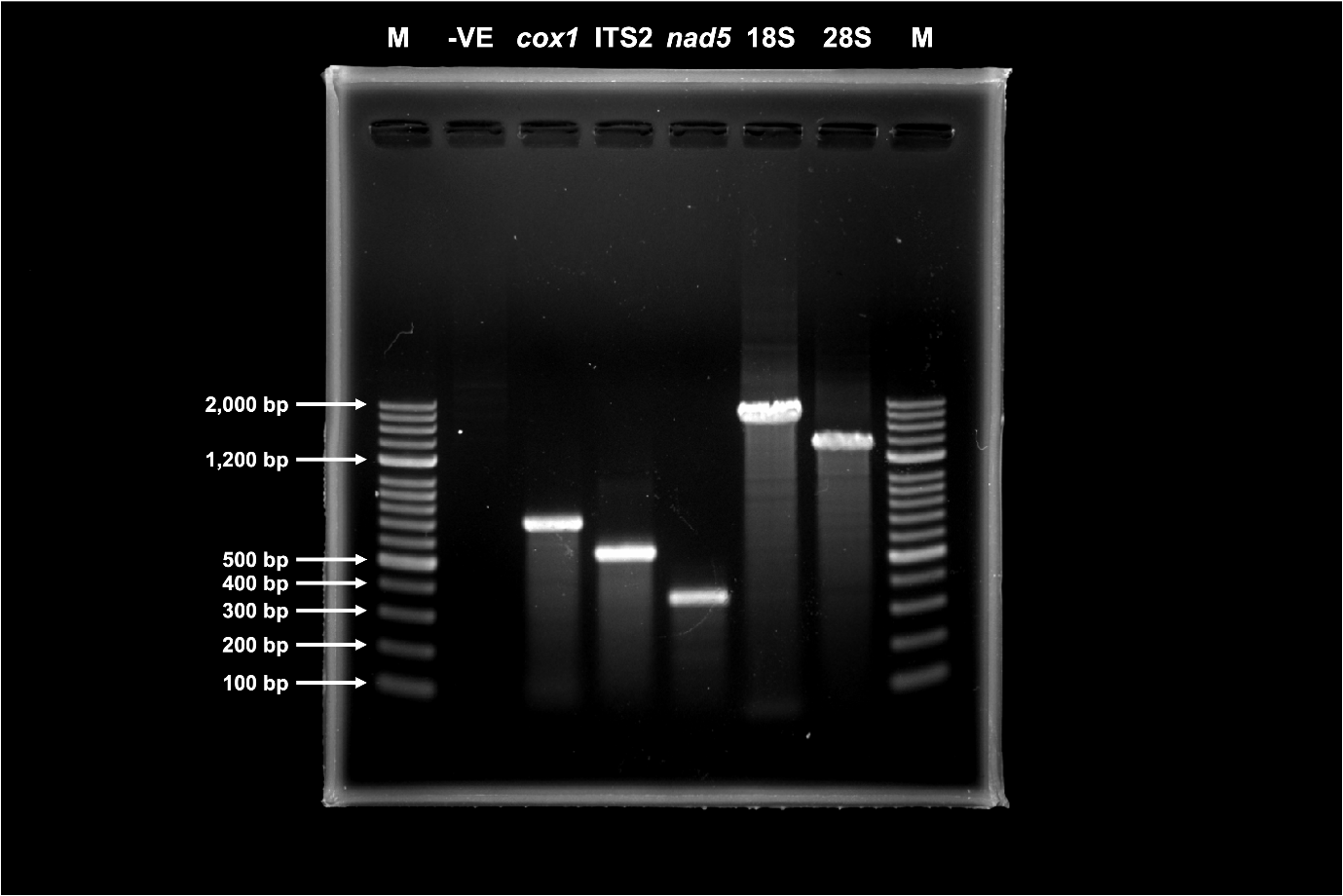


**Fig. S1** Polymerase chain reaction products of *cox1*, ITS2, *nad5*, 18S rRNA, and 28S rRNA genes for pentastomid species identification. Lanes 1 through 8 are, from left to right, 100-bp ladder marker (M), negative control (-VE), *cox1* (707 bp), ITS2 (544 bp), *nad5* (358 bp), 18S rRNA (18S, 1842 bp), 28S rRNA (28S, 1367 bp), and 100-bp ladder marker (M)
