# Supplementary material for: First complete mitochondrial genome of Armillifer moniliformis (Pentastomida: Porocephalida) isolated from a human case in Northern Thailand: comparative and phylogenetic analyses
Source: Parasitol Res. 2025 Jun 27;124(6):69. doi: 10.1007/s00436-025-08516-x (PMC12202648; doi:10.1007/s00436-025-08516-x)
Supplement: Supplementary file 5 — Supplementary file5 (DOCX 28.1 KB) [file 436_2025_8516_MOESM5_ESM.docx]

**Table S4** BLASTn results of mitochondrial and nuclear gene sequences obtained from pentastomid gDNA samples in this study

| Gene location | Gene name | Amplicon size (bp) | Accession no. | Top BLASTn result with accession no. | Query coverage | % Identity | E-value |
| --- | --- | --- | --- | --- | --- | --- | --- |
| Mitochondrial | *cox1* | 707 | PP272417 | *Armillifer moniliformis* voucher SW-2019 (MN531845) | 91% | 97.7% | 0 |
|  | *nad5* | 358 | PP273415 | *Armillifer* sp. SW-2019 (MK063889) | 84% | 98.3% | 4e-146 |
| Nuclear | ITS2 | 544 | PP273236 | *Armillifer* sp. SW-2019 (MK063888) | 70% | 100% | 0 |
|  | 18S rRNA | 1,842 | PV124096 | *Armillifer moniliformis* isolate Yunnan (ON982766) | 100% | 99.4% | 0 |
|  | 28S rRNA | 1,367 | PV132650 | *Porocephalus* sp. JG2020 (MT387200) | 100% | 86.5% | 0 |
