# Supplementary material for: First complete mitochondrial genome of Armillifer moniliformis (Pentastomida: Porocephalida) isolated from a human case in Northern Thailand: comparative and phylogenetic analyses
Source: Parasitol Res. 2025 Jun 27;124(6):69. doi: 10.1007/s00436-025-08516-x (PMC12202648; doi:10.1007/s00436-025-08516-x)
Supplement: Supplementary file 6 — Supplementary file6 (DOCX 27 KB) [file 436_2025_8516_MOESM6_ESM.docx]

**Table S5** List of the best-performing substitution models for each partition in phylogenetic analysis using the full concatenation approach

| Gene/partition | Substitution model |
| --- | --- |
| *nad2* | TVM+F+I+G4 |
| *cox1* | GTR+F+I+R3 |
| *cox2* | GTR+F+I+R4 |
| *atp8* | TVM+F+I+G4 |
| *atp6* | GTR+F+I+R4 |
| *cox3* | GTR+F+I+R4 |
| *nad3* | GTR+F+I+R4 |
| *nad5* | GTR+F+R5 |
| *nad4* | GTR+F+R5 |
| *nad4L* | GTR+F+R5 |
| *nad6* | GTR+F+I+R4 |
| *cytb* | GTR+F+I+R4 |
| *nad1* | GTR+F+R5 |

TVM: Transversion model

GTR: General time reversible model with unequal rates and unequal base frequency

+F: Empirical base frequencies

+I: Invariable site

+G: Discrete Gamma model

+R: FreeRate model
