# Supplementary material for: First complete mitochondrial genome of Armillifer moniliformis (Pentastomida: Porocephalida) isolated from a human case in Northern Thailand: comparative and phylogenetic analyses
Source: Parasitol Res. 2025 Jun 27;124(6):69. doi: 10.1007/s00436-025-08516-x (PMC12202648; doi:10.1007/s00436-025-08516-x)
Supplement: Supplementary file 7 — Supplementary file7 (DOCX 27.6 KB) [file 436_2025_8516_MOESM7_ESM.docx]

**Table S6** Pairwise genetic similarity of the longest repetitive NCR segment across *A. moniliformis* from this study and the other three *Armillifer* species that have previously been sequenced

| Species | *Armillifer armillatus* | *Armillifer grandis* | *Armillifer agkistrodontis* | *Armillifer moniliformis* |
| --- | --- | --- | --- | --- |
| *Armillifer armillatus* |  |  |  |  |
| *Armillifer grandis* | 0.786 |  |  |  |
| *Armillifer agkistrodontis* | 0.739 | 0.729 |  |  |
| *Armillifer moniliformis* | 0.594 | 0.620 | 0.640 |  |

N.B. The longest repetitive NCR segments in *Armillifer armillatus* (AY456186), *A. grandis* (KY914472), *A. agkistrodontis* (KX686568), and *A. moniliformis* (PV138266) are 1904 bp, 1839 bp, 1872 bp, and 1659 bp. long, respectively
