# Supplementary material for: First complete mitochondrial genome of Armillifer moniliformis (Pentastomida: Porocephalida) isolated from a human case in Northern Thailand: comparative and phylogenetic analyses
Source: Parasitol Res. 2025 Jun 27;124(6):69. doi: 10.1007/s00436-025-08516-x (PMC12202648; doi:10.1007/s00436-025-08516-x)

**Fig. S2** Entropy plot showing sequence polymorphism of the longest repetitive NCR segments, consisting of five tandem repeats, across four *Armillifer* species


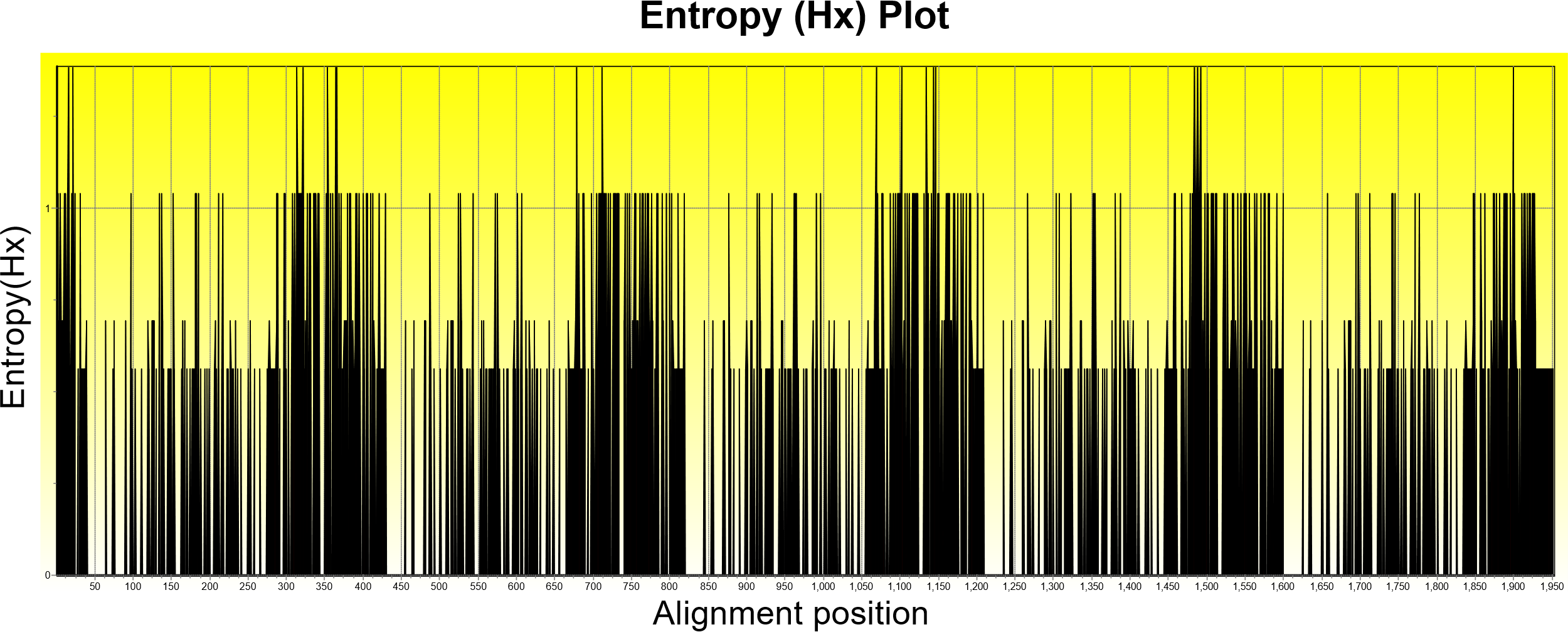

Supplement: Supplementary file 8 — Supplementary file8 (DOCX 896 KB) [file 436_2025_8516_MOESM8_ESM.docx]
