# Supplementary material for: First complete mitochondrial genome of Armillifer moniliformis (Pentastomida: Porocephalida) isolated from a human case in Northern Thailand: comparative and phylogenetic analyses
Source: Parasitol Res. 2025 Jun 27;124(6):69. doi: 10.1007/s00436-025-08516-x (PMC12202648; doi:10.1007/s00436-025-08516-x)
Supplement: Supplementary file 9 — Supplementary file9 (DOCX 537 KB) [file 436_2025_8516_MOESM9_ESM.docx]

**Fig. S3** Phylogenetic relationships of *A. moniliformis*, other pentastomids, and other arthropod groups based on *nad4* (**A**) and *nad5* (**B**) sequences using the maximum likelihood method, showing different topologies between the resulting trees. *Hypsibius dujardini* was used as an outgroup
